# Evaluation study of radial and spiral based volumetric thermometry for monitoring of hepatic microwave ablation

**DOI:** 10.1038/s41598-025-20588-4

**Published:** 2025-09-24

**Authors:** Dominik Horstmann, Othmar Belker, Daniel Düx, Thomas Gerlach, Moritz Gutt, Simon Schröer, Ivan Vogt, Frank Wacker, Bennet Hensen, Marcel Gutberlet

**Affiliations:** 1 Department of Diagnostic and Interventional Radiology, Hannover Medical School, Carl-Neuberg-Str. 1, 30625 Hannover, Germany; 2 Research Campus STIMULATE Magdeburg, Magdeburg, Germany; 3 Department Biomedical Magnetic Resonance, Otto-von-Guericke University, Magdeburg, Germany

**Keywords:** Stack-of-stars, Stack-of-spirals, 3D MR thermometry, Respiratory motion, Microwave ablation, Imaging techniques, Translational research

## Abstract

Microwave ablation (MWA) of hepatic tumors benefits from MR thermometry, enabling real-time temperature monitoring to guide treatment and protect healthy tissue. However, MR thermometry in the abdomen is challenging due to respiratory and intestinal motion. This study evaluates two advanced 3D imaging sequences, Stack-of-Stars (Stars) and Stack-of-Spirals (Spirals), for precise MWA thermometry in phantom and volunteer experiments. Spirals demonstrated superior temperature precision, with a standard deviation of 1.61 ± 0.11 °C in unheated regions, compared to 4.87 ± 0.74 °C for Stars (single respiratory cycle). In heated regions, Spirals achieved a lower RMSE (0.6 ± 0.1 °C vs. 1.3 ± 0.3 °C for Stars) and a higher Dice score for ablation zone delineation (0.88 ± 0.02 vs. 0.77 ± 0.09). Spirals also produced sharper images with fewer artifacts under simulated respiratory motion, while Stars showed streaking artifacts due to higher undersampling. These findings highlight Spirals’ potential for accurate real-time thermometry in liver ablation. Future work should focus on improving reconstruction speed and mitigating susceptibility artifacts to enable clinical applications.

**Supplementary Information:**

The online version contains supplementary material available at 10.1038/s41598-025-20588-4

## Introduction

Microwave ablation (MWA) is a widely used thermal therapy that destroys tumor tissues by applying heat over larger areas compared to other modalities^1^. Unlike ultrasound and computed tomography (CT), magnetic resonance imaging (MRI) offers real-time monitoring of temperature changes during procedures through MR thermometry, making it a valuable tool for precise treatment guidance^2^.

Precise temperature monitoring during ablation is crucial for assessing tissue damage and guiding decisions on treatment continuation, duration, and targeting. MR thermometry has been shown to predict post-procedural ablation zones accurately, enabling real-time evaluation of treatment outcomes. This capability reduces recurrence risk by allowing immediate re-ablation of under-treated areas and protects nearby sensitive structures, such as the bowel^3,4^.

In hepatic microwave ablation, thermal lesions typically do not exceed 5 cm in size, requiring volumetric coverage of approximately 6 cm to ensure complete thermal monitoring^5^. Given typical ablation durations of 5–10 min, robust and continuous thermometry over this volume is essential to guide and assess treatment.

Among MR-based techniques, proton resonance frequency shift (PRFS) thermometry is widely used in clinical applications due to its linear temperature dependence and minimal sensitivity to tissue-specific variations, except in adipose tissue^6^. By leveraging phase variations in conventional MRI sequences, PRFS thermometry achieves high spatial and temporal resolution, making it suitable for real-time monitoring during thermal therapies. However, it remains susceptible to motion, phase drift, and susceptibility changes induced by temperature fluctuations, tissue transitions, or gas formation during ablation^7–9^.

For PRFS thermometry to be clinically effective, it must combine motion robustness, high sensitivity, and minimal susceptibility to artifacts, while maintaining sufficient spatial and temporal resolution^10^. Achieving this balance remains a major challenge in abdominal imaging.

Two-dimensional SMS-EPI thermometry has demonstrated excellent temporal and spatial resolution, enabling volumetric coverage within a single respiratory cycle^11^. In well-controlled clinical settings—such as under anesthesia during hepatic microwave ablation—this makes it a powerful and widely adopted tool for MR thermometry.

Complementary to this, 3D thermometry approaches offer distinct advantages: by exciting a contiguous slab rather than a series of discrete slices, they enable intrinsically higher signal-to-noise efficiency and eliminate the need for slice planning by acquiring a contiguous volume. However, the extended acquisition times required for 3D imaging necessitate aggressive undersampling, which poses challenges to temperature accuracy and dynamic fidelity unless mitigated by robust reconstruction techniques.

This work investigates two 3D non-Cartesian sequences—Stack-of-Spirals (Spirals) and Stack-of-Stars (Stars)—that leverage incoherent aliasing and compressed sensing to enable highly undersampled volumetric thermometry. While Spirals provide efficient k-space sampling for higher spatiotemporal resolution, Stars may offer increased robustness to B₀ inhomogeneities and T₂*-related signal loss due to their radial acquisition strategy and associated shorter effective echo times^12–14^. This study evaluates the clinical relevance of both approaches in phantom and volunteer experiments focusing on temperature stability, the influence of undersampling on reconstruction accuracy, and the quantitative assessment of ablation zone delineation under realistic conditions.

## Results

### Phantom study: analysis of magnitude images and undersampling

On average, the Stack-of-Stars (Stars) sequence utilized 13 spokes per breath, each with 8 partitions across 7 echoes, while the Stack-of-Spirals (Spirals) sequence employed 7 interleaves per breath, each with 12 partitions and 2 echoes. For a 480 mm field of view (FOV), as used for the phantoms, the resulting undersampling factors ranged from 8.6 (1 breathing cycle, nBC1) to 2.4 (2 breathing cycles, nBC2) for Spirals and from 69.5 (nBC1) to 34.8 (nBC2) for Stars, where nBC denotes the number of breathing cycles per reconstructed image.

**Fig. 1:**
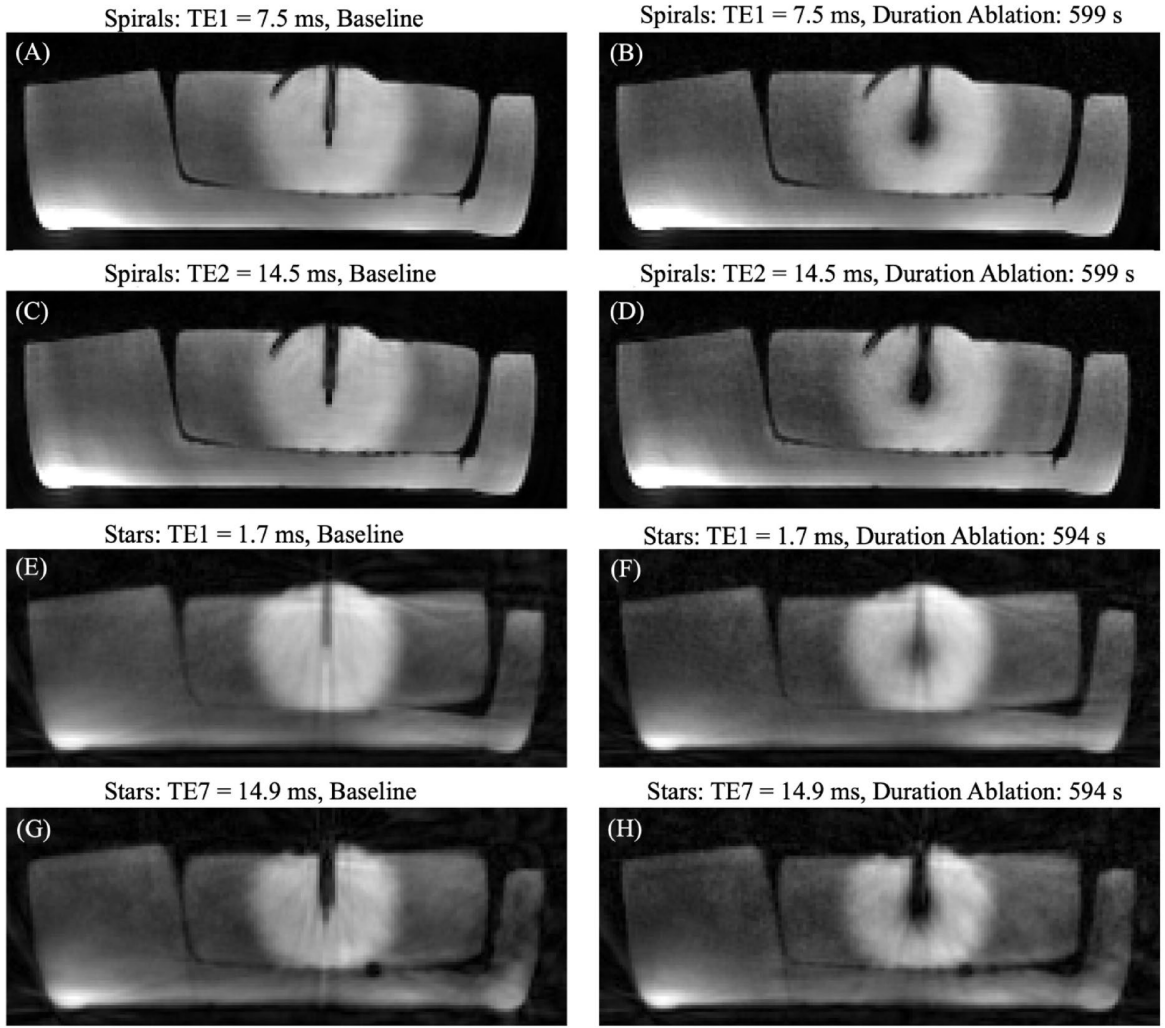
Magnitude images of the phantom during simulated breathing. (**A-D**) Magnitude images of the phantom acquired with the Stack-of-Spirals and (**E-H**) Stack-of-Stars sequence during simulated breathing with temporal resolution of two breathing cycles (nBC2) during baseline (**A**,** C** and **E**,** G**) and shortly before the end (**B**,** D** and **F**,** H**) of 10 min ablation. For both Spirals and Stars, the first (TE = 7.5 ms/1.7 ms) (1st and 3rd row) and last echo (TE = 14.7 ms/14.9 ms) (2nd and 4th row) is given.

These differences in undersampling were reflected in the image quality as shown in Fig. 1, which compares magnitude images from Spirals (Fig. 1A-D) and Stars (Fig. 1E-H) at the smallest and largest echo times (TE). Images acquired with Stars exhibited noticeable streaking artifacts caused by more aggressive undersampling, while those obtained with Spirals were largely free of artifacts, demonstrating cleaner and more consistent quality.

As TE increased, signal loss around the needle due to T2* effects became more pronounced, enlarging the needle artifact. This effect was further exacerbated by heating, leading to additional signal loss. Due to its shorter minimum TE of 1.7 ms, Stars exhibited a smaller needle artifact and less signal loss at baseline and during heating compared to Spirals.

### Temperature precision in unheated regions

Temperature accuracy in unheated regions differed significantly across all examined nBCs. The Stack-of-Spirals (Spirals) sequence consistently exhibited superior temperature precision, with mean temperature standard deviations ranging from 1.08 ± 0.09 °C (nBC2) to 1.61 ± 0.11 °C (nBC1). In comparison, the Stack-of-Stars (Stars) sequence showed higher mean temperature standard deviations, ranging from 3.43 ± 0.23 °C (nBC2) to 4.87 ± 0.74 °C (nBC1).

Differences between nBC1 and nBC2 were significant for both Spirals (*p* < 0.0001) and Stars (*p* = 0.0008).

### Temperature precision in heated regions

**Fig. 2:**
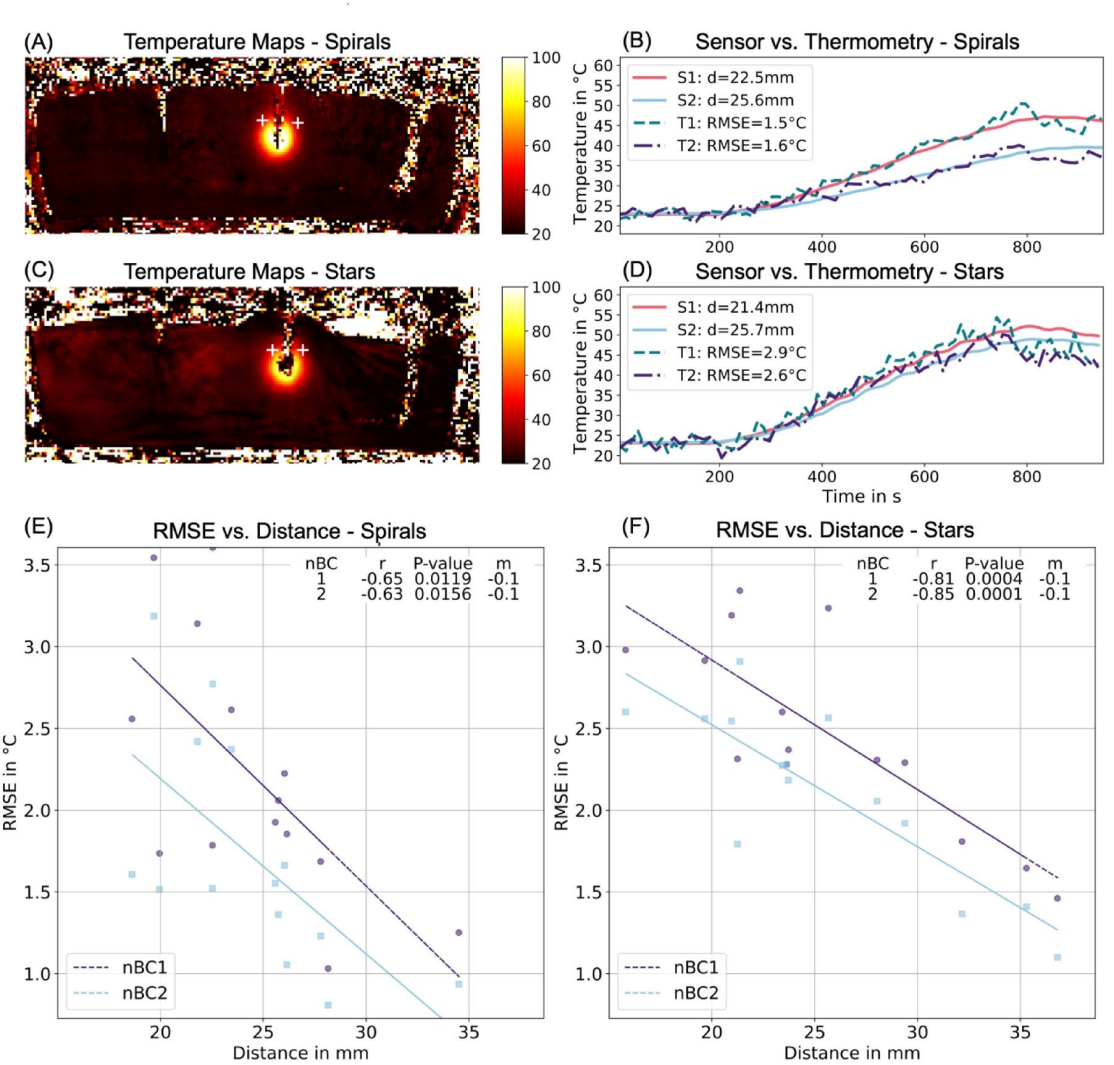
Temperature precision in heated areas. Transversal slice from a 3D temperature map acquired with the Stack-of-Spirals **(A)** and Stack-of-Stars **(C)** sequence over two breathing cycles (nBC2), including both temperature sensors (marked by white crosses), captured shortly before the end of the 10-minute ablation. Temperature profiles for the two sensors (S1/S2) in solid lines and the corresponding thermometry (T1/T2) in dotted lines for Spirals **(B)** and Stars **(D)**. Distance d of the sensors to the center of the ablation zone and root Mean Squared Error (RMSE) between the sensors and thermometry are indicated in the legend. Scatter plots of RMSE results dependent on d for each nBC using Spirals **(E)** and Stars **(F)**. Linear regression lines are fitted to the data, with correlation coefficient (r), significance (p-value), and slope (m) in °C/mm detailed in the legend.

Figure 2 (A-D) presents representative transverse slices from 3D temperature maps and corresponding temperature profiles from a phantom during ablation using Stars and Spirals, compared to data from the temperature sensors. Scatter plots (Fig. 2E-F) depict the relationship between Root Mean Squared Error (RMSE) and the distance from the ablation center (), revealing that temperature accuracy, represented by RMSE, improves with increasing distance from the ablation center, a trend seen across all nBCs. Notably, the linear regression fits are significant. The regression lines for Spirals consistently lie below those for Stars, indicating superior temperature accuracy in heated regions for Spirals.

The Analysis of Covariance (ANCOVA) results confirmed significant differences in RMSE between Stars and Spirals for nBC2 (F = 11.06, *p* = 0.0025) supporting Spirals’ superior temperature accuracy across these conditions. The distance from the ablation center significantly influenced RMSE across all nBCs (e.g., F = 29.22, *p* < 0.0001 for nBC2), highlighting its critical role in temperature measurement accuracy.

The interaction term between method and distance was not significant for any nBC (e.g., F = 0.13, *p* = 0.7180 for nBC2), suggesting that the RMSE-distance relationship remained consistent across both methods. Analysis of Variance (ANOVA) showed a significant effect on RMSE for Spirals (F = 3.91, *p* = 0.013), with Tukey’s HSD revealing a significant difference between nBC1 and nBC2 for Spirals whereas no significant effect was found for Stars (F = 1.12, *p* = 0.349).

### Precision of the calculated ablation zone

**Fig. 3:**
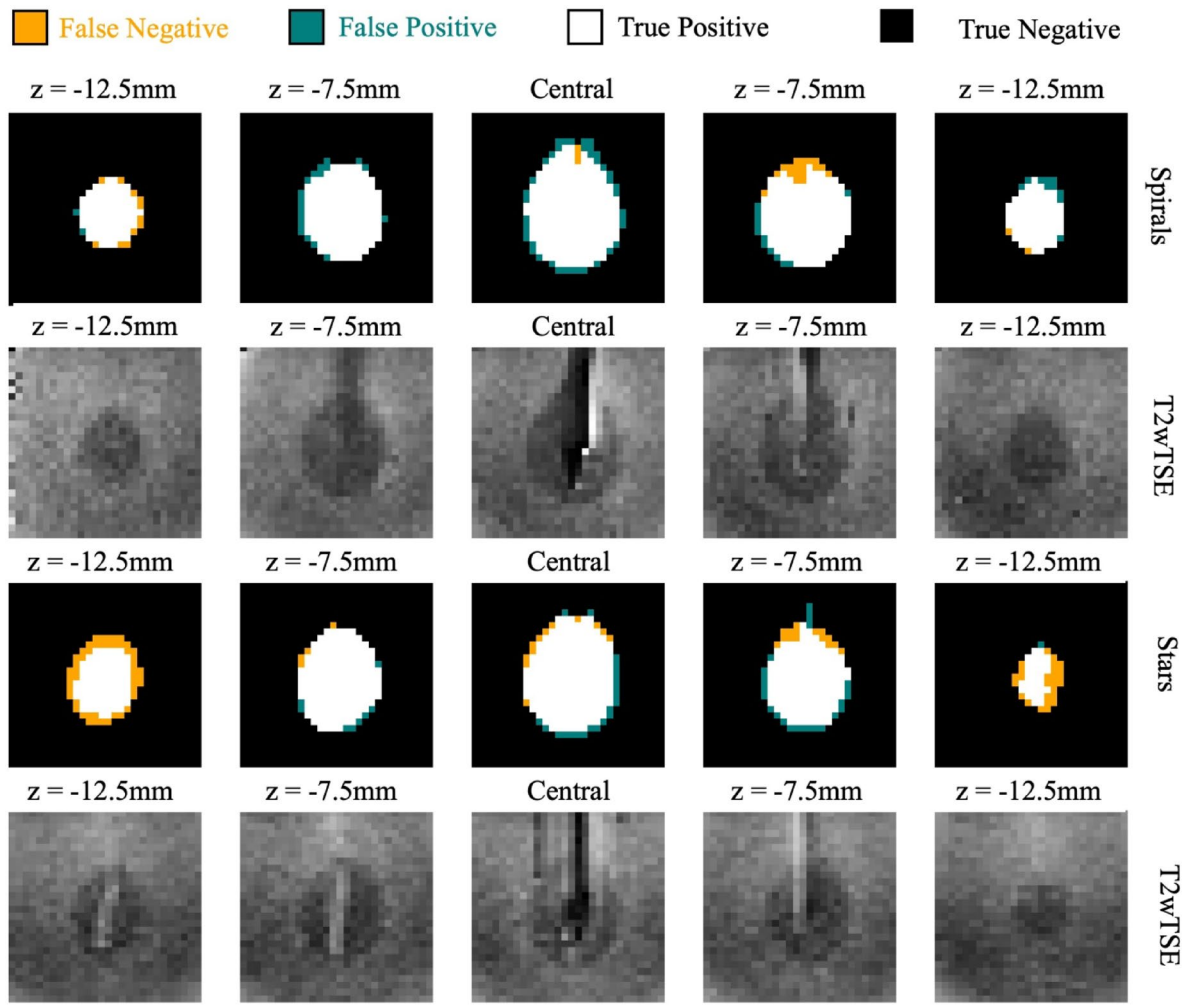
Calculated ablation zones overlaid with ground truth. Transverse slices of the ablation zones calculated from MR thermometry using Spirals (first row) and Stars (third row) over two breathing cycles (nBC2) are overlaid with the ground truth derived from T2-weighted TSE images (second and forth row). White, orange, cyan, and black voxels represent true positives, false negatives, false positives, and true negatives, respectively. Ground truth segmentations were initially performed on the native high-resolution T2wTSE data and then transformed using interpolation and co-registration **between** T2wTSE images and MR thermometry magnitude images.

Figure 3 illustrates the comparison between the calculated ablation zones and the ground truth for a representative phantom. Both Stars and Spirals demonstrated close alignment with the ground truth. However, near the ablation center, Spirals exhibited more false positives than Stars, indicating a slight overestimation of the ablation zone. Conversely, in peripheral regions, particularly for Stars, false negatives were more prevalent, reflecting an underestimation of the ablation zone boundaries.

**Table 1:** Metrics comparing ablation zone accuracy between Stack-of-Stars and Stack-of-Spirals across temporal resolutions.

Metric	Method	nBC1		nBC2	
		µ ± σ	ES	µ ± σ	ES
Dice	Spirals	0.88 ± 0.02	1.73	0.89 ± 0.01	1.83
	Stars	0.77 ± 0.09		0.77 ± 0.10	
MSD	Spirals	0.13 ± 0.02	−1.51	0.11 ± 0.02	−1.61
	Stars	0.33 ± 0.19		0.34 ± 0.20	
Sensitivity	Spirals	0.91 ± 0.03	1.47	0.91 ± 0.03	1.53
	Stars	0.75 ± 0.15		0.73 ± 0.16	

Dice = Dice Score, ES = Effect Size, MSD = Mean Surface Distance, µ = Mean Value, nBC = Number of Breathing Cycles, σ = Standard Deviation, Spirals = Stack-of-Spirals, Stars = Stack-of-Stars.

This observation is corroborated by the statistical metrics presented in Table 1. Across both nBCs, Spirals consistently outperformed Stars, as evidenced by higher Dice scores, lower Mean Surface Differences (MSD), and greater Sensitivity values, confirming Spirals’ superior accuracy in delineating the ablation zone. All differences were statistically significant, with large effect sizes.

ANOVA results revealed no significant differences across nBCs for any metric (Dice, MSD, Sensitivity) in either Spirals or Stars, suggesting that temporal resolution did not affect the accuracy of ablation zone measurements. Consequently, no further post hoc comparisons were performed.

### Volunteer study

**Fig. 4:**
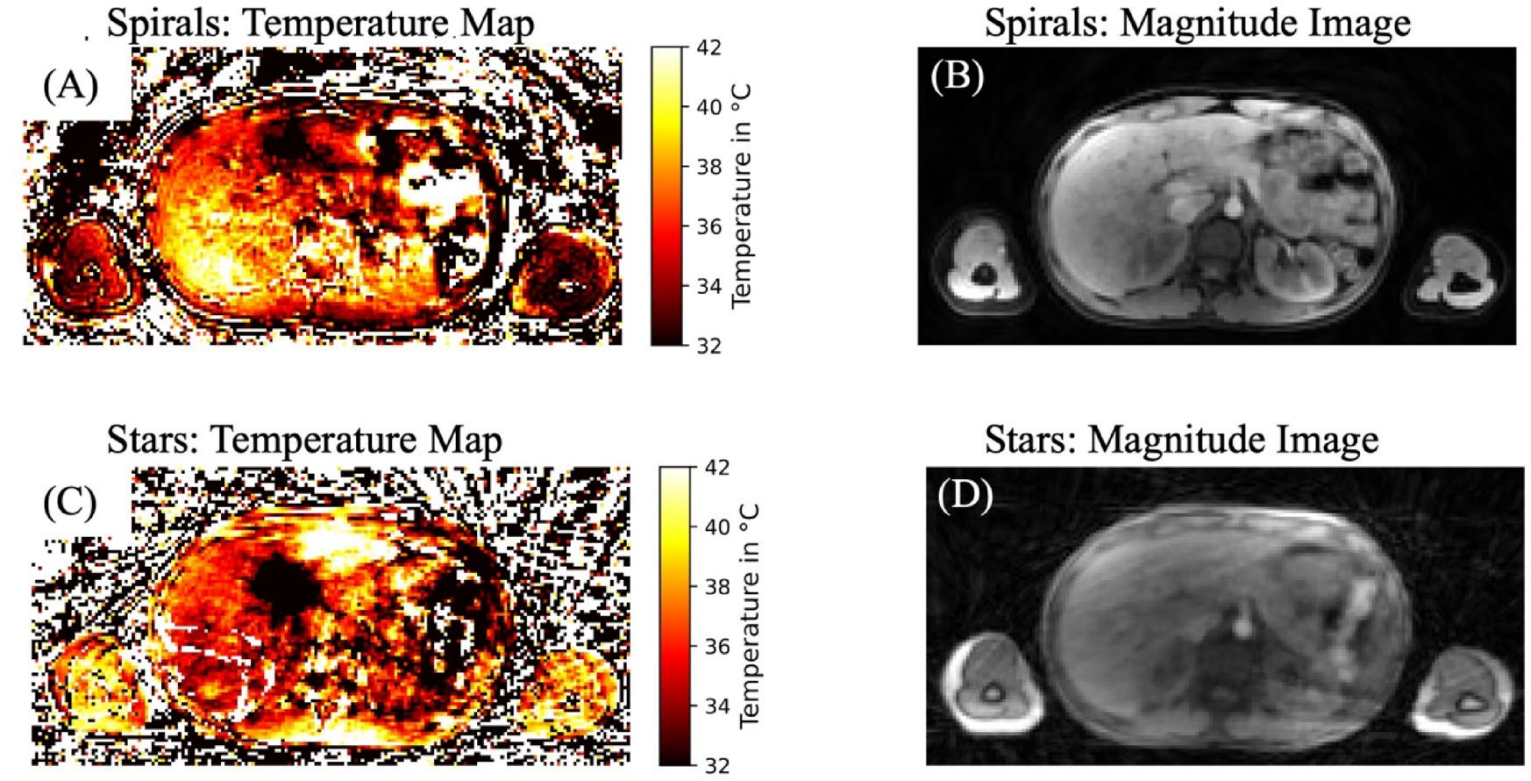
Magnitude images and temperature maps of a volunteer. Transverse slice of the temperature map **(A**,** C)** and corresponding magnitude image of the first echo **(B**,** D)** from a volunteer acquired with the Stack-of-Spirals (**A**, **B**; 1 st row) and Stack-of-Stars (**C**, **D**; 2nd row) sequences at a temporal resolution of two breathing cycles (nBC2). No ablation was performed. The temperature maps reflect baseline fluctuations due to measurement noise and physiological motion rather than actual heating, assuming a baseline of 36.5 °C.

Figure 4 presents representative temperature maps for a transverse slice acquired with (Fig. 4A) Spirals and (Fig. 4B) Stars, alongside corresponding magnitude images (Fig. 4. C and D). Both sequences exhibited significant susceptibility artifacts in the temperature maps, primarily due to bowel motion. As no ablation was performed, the volunteer temperature maps reflect baseline fluctuations from measurement noise, susceptibility, and physiological motion rather than true heating, assuming a baseline temperature of 36.5 °C. The magnitude images for Stars displayed more pronounced artifacts, whereas Spirals images provided enhanced anatomical detail, including sharper visualization of vessels and reduced blurriness. The effectiveness of fat saturation in Spirals was evident, with subcutaneous fat—bright in Stars—exhibiting minimal signal in Spirals.

**Table 2:** Temperature standard deviation in liver regions across temporal resolutions with statistical comparisons of Stack-of-Stars and Stack-of-Spirals.

nBC	Method	Caudal			Middle			Cranial		
		µ ± σ [°C]	p-value	ES	µ ± σ [°C]	p-value	ES	µ ± σ [°C]	p-value	ES
1	Spirals	2.5 ± 1.3	0.175	−0.42	2.7 ± 1.4	0.193	−0.40	2.9 ± 1.3	0.189	−0.41
	Stars	3.3 ± 0.9			3.5 ± 0.9			4.0 ± 1.8		
2	Spirals	1.9 ± 1.1	0.048	−0.58	2.1 ± 1.0	0.051	−0.57	2.1 ± 0.9	0.042	−0.59
	Stars	2.9 ± 0.8			3.1 ± 1.0			3.7 ± 1.7		

ES = Effect Sizes, µ = Mean Value of Temperature Standard Deviation over Time, nBC = Number of Breathing Cycles, σ = Standard Deviation of Temperature Standard Deviation over Time, Spirals = Stack-of-Spirals, Stars = Stack-of-Stars.

Table 2 summarizes the temperature standard deviation (STD) across caudal, middle, and cranial liver regions for Spirals and Stars at nBC1 and nBC2. While no statistically significant differences were observed for nBC1, Spirals exhibited consistently lower mean STDs across all regions. At nBC2, these differences became statistically significant in the caudal (*p* = 0.048) and cranial (*p* = 0.042) regions, and approached significance in the middle region (*p* = 0.051). These findings suggest improved temperature stability of Spiral acquisitions.

### Reconstruction time

The reconstruction times for one 3D image for Spirals were 35 s (nBC2) and 33 s (nBC1), and for Stars 42 s (nBC2) and 40 s (nBC1).

## Discussion

This study demonstrated that the Stack-of-Spirals sequence consistently outperformed the Stack-of-Stars sequence in 3D PRFS-based MR thermometry. Key advantages of Spirals included superior temperature precision, lower root mean squared error (RMSE) in heated regions, and more accurate delineation of ablation zones, reflected by higher Dice scores, lower Mean Surface Distance (MSD), and greater sensitivity values. These findings were consistent across phantom and volunteer experiments. This establishes the Stack-of-Spirals sequence as a promising approach for clinical applications of MR thermometry, particularly in hepatic microwave ablation.

The findings of this study align with prior research on MR thermometry: Marx et al. achieved temperature accuracies below 0.5 °C in the brain with spiral sequences and high spatial resolution (< 1.5 mm)^14^. While these results demonstrate excellent precision, they were achieved in static 2D brain imaging, a simpler context compared to the challenges of 3D imaging in a moving liver. Similarly, Kim et al. reported liver temperature accuracies of 1–2 °C using a multi-baseline strategy in 2D imaging, suggesting potential improvements for 3D imaging if such methods are adapted^15^. Their limited 2D coverage (three 5-mm slices) underscores the advantage of the 60-mm volumetric coverage in this study for larger ablation zones.

Dietrich et al. achieved sub-1 °C precision using an EPI sequence across 25 slices in static phantom experiments^16^. While their findings provide valuable insights, the absence of motion artifacts in their setup limits their clinical relevance. Ozenne et al. demonstrated that SMS-EPI can achieve excellent spatiotemporal resolution for 2D thermometry, reporting temperature accuracies of approximately 2 °C in unheated regions and below 1 °C in phantom ablation zones^11^. These results underscore the capabilities of 2D SMS-EPI in providing high temporal fidelity and precision.

Our findings complement this perspective by demonstrating that 3D thermometry can serve as a viable alternative in scenarios requiring full volumetric coverage. Despite inherently longer acquisition windows, we show that volumetric temperature maps with high accuracy can be acquired within one respiratory cycle—consistent with clinically acceptable timeframes. In addition to enabling uniform slab excitation, 3D acquisitions offer high SNR efficiency and increased flexibility in spatial planning, which facilitates comprehensive assessment of thermal spread and reduces the risk of overlooking clinically relevant heating patterns. Moreover, the relatively short readout durations used in our 3D non-Cartesian implementations of less than 7 ms guarantee resilience to susceptibility-related artifacts.

Overall, this study builds upon existing literature by demonstrating a robust 3D thermometry approach that offers comparable accuracy to 2D methods while addressing motion and susceptibility challenges in the liver.

While this study explored both single-cycle (nBC1) and extended (nBC2) acquisition windows, our results show that nBC1 reconstructions already provide robust temperature stability and accurate ablation zone delineation. Given that nBC1 corresponds to a 5-second acquisition window—well within the temporal constraints recommended for clinical MR thermometry—it emerges as the more relevant condition for real-time monitoring. Extended acquisition (nBC2) offered only marginal improvements in temperature precision at the cost of delayed temperature updates, while potentially introducing temporal smoothing. These findings underscore that increasing temporal resolution beyond a single respiratory cycle does not necessarily improve thermometric performance and may compromise the dynamic fidelity required during ablation.

The application of compressed sensing with temporal regularization in PRFS-based thermometry has been previously validated in related contexts, notably by Todd et al.^17^, who demonstrated accurate and real-time 3D temperature mapping during high-intensity focused ultrasound (HIFU) using temporally constrained reconstruction. Our study builds upon this foundation by implementing a similar temporal regularization strategy within a non-Cartesian acquisition and reconstruction pipeline, thereby extending these principles to hepatic MWA. To evaluate thermometric accuracy under realistic conditions, we relied on experimental validation using fiber-optic temperature probes, RMSE analysis at varying distances from the applicator, and comparison to post-ablation reference imaging. This approach captures key error sources such as motion, susceptibility, and signal variability, and reflects the practical performance of the reconstruction pipeline. While synthetic validation may offer additional insights into temporal fidelity—particularly for specific reconstruction behaviors—it requires extensive modeling of physiological, technical and imaging parameters. Future studies could complement experimental validation with realistic simulation data to further refine temperature accuracy assessments.

Despite these promising results, the study also faces notable limitations. Reconstruction times of 33–40 s per 3D image are currently insufficient for real-time clinical application, mainly due to the reliance on a single GPU and the lack of an efficient implementation of temporal regularization that reuses previously reconstructed images. Future work will focus on optimizing GPU utilization and introducing more efficient reconstruction algorithms. Additionally, susceptibility artifacts, particularly near air-tissue interfaces and in the presence of gas bubbles during ablation, remain a challenge. Incorporating dedicated susceptibility correction methods could further improve thermometric accuracy in these regions^18–21^. Furthermore, the volunteer study was conducted under free-breathing conditions without thermal treatment, which may not fully capture the complexities of clinical ablation scenarios.

Future work should aim to translate the demonstrated feasibility of Stack-of-Spirals thermometry into clinical practice. This includes implementing real-time reconstruction pipelines through optimized GPU usage and algorithmic acceleration, potentially aided by machine learning techniques. Additionally, strategies to increase robustness in anatomically challenging regions—such as referenceless thermometry, improved motion correction, and multi-baseline methods—should be integrated to handle susceptibility-related distortions and motion near the diaphragm or bowel^15^. Most importantly, clinical validation during microwave ablation procedures will be essential to evaluate the reliability of this method in guiding real-time treatment decisions.

Taken together, these findings demonstrate that Stack-of-Spirals thermometry provides a robust and accurate 3D solution for PRFS-based temperature monitoring with full tumor coverage. Its ability to achieve sub-3 °C temperature accuracy and high spatial agreement within a single respiratory cycle underscores its clinical potential as a volumetric alternative to conventional 2D thermometry techniques.

## Methods

This study was approved by the Institutional Review Board (IRB) of Hannover Medical School (Approval No. 11019_B0_S_2023) and was conducted in accordance with the principles of the Declaration of Helsinki and relevant national and institutional guidelines. All procedures involving human participants were performed in accordance with the ethical standards of the Hannover Medical School Ethics Committee and the 1964 Helsinki Declaration and its later amendments. Written informed consent was obtained from all participants prior to their inclusion in the study.

The aim was to evaluate the performance of Stack-of-Stars (Stars) and Stack-of-Spirals (Spirals) sequences in abdominal 3D PRFS-based MR thermometry. Phantom experiments simulated controlled conditions, while volunteer experiments assessed sequence performance under free-breathing conditions.

### Sequence implementation

**Fig. 5:**
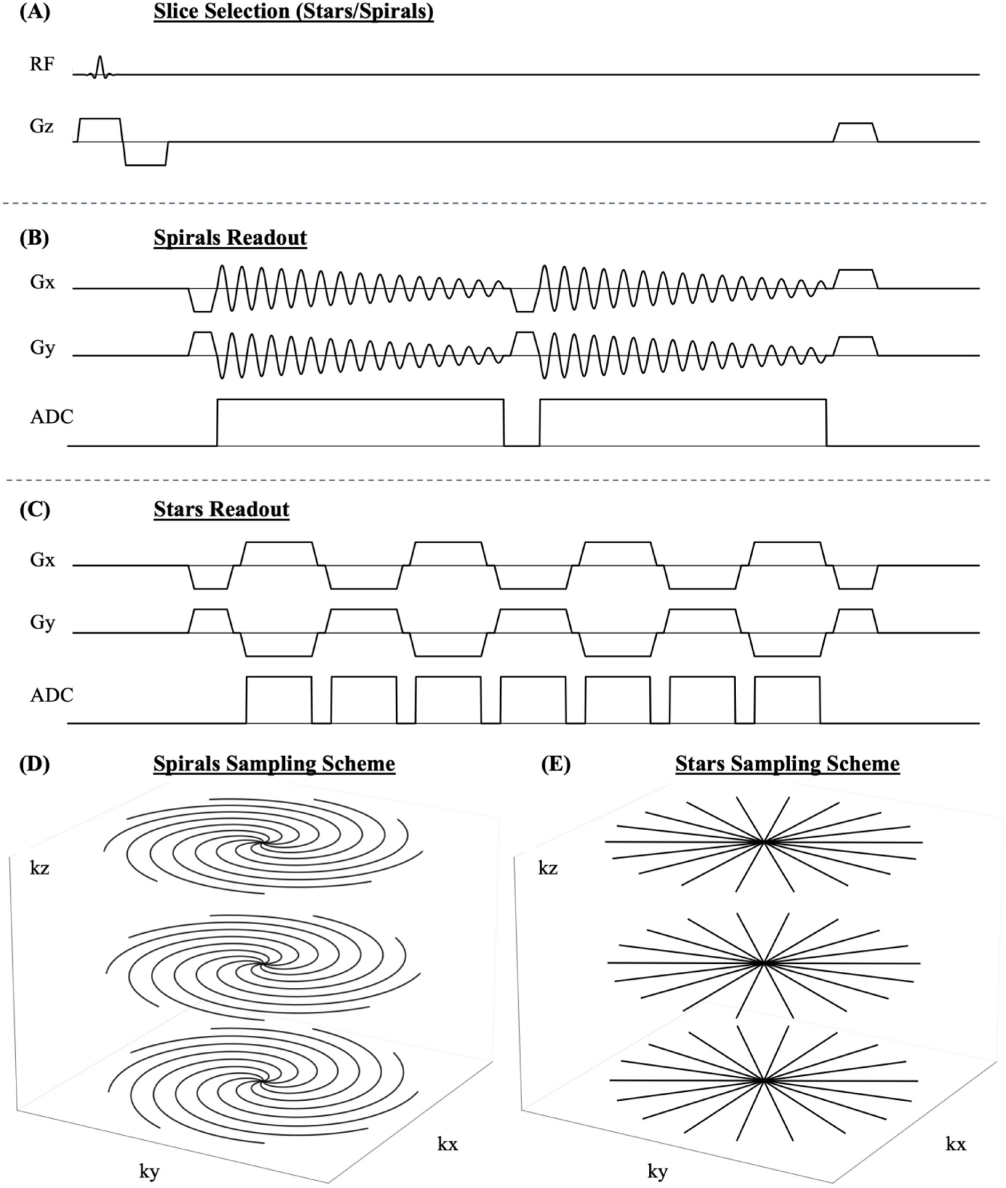
Sequence diagram. **(A)** Slab-selective excitation with Cartesian sampling in z-direction and **(B)** either spiral-in spiral-in readout for Spirals or **(C)** seven consecutive echoes with linear sampling using bipolar gradients for Stars in x-y-plane. An excerpt of the resulting Stack-of-Spirals and Stack-of-Stars in 3D k-space is shown in **(D)** and **(E)**.

Figure 5 illustrates the sequence diagrams for the Stack-of-Stars (Stars) and Stack-of-Spirals (Spirals) implementations. Stars was realized as a multi-echo spoiled gradient echo with a 4th-order tiny golden angle (38.98°) increment between projections^22^. Projections were rotated after completing Cartesian kz-sampling. The sequence used seven bipolar echoes with alternating gradient polarity per readout, featuring echo times (TE) from 1.7 ms to 14.9 ms and a repetition time (TR) of 17.3 ms. Other parameters included a field of view (FOV) of 480 × 480 × 60 mm³, a spatial resolution of 2.5 × 2.5 × 2.5 mm³, and a bandwidth of 520 Hz/pixel.

For the Stack-of-Spirals (Spirals) sequence, a dual-echo spiral-in/spiral-in spoiled gradient echo was implemented with a 12th-order tiny golden angle increment (14.27°). The sequence parameters were TE = 7.5 ms/14.5 ms, TR = 22.6 ms, and a bandwidth of 1040 Hz/pixel. Spiral rotation was applied after completing Cartesian kz-sampling. A variable-density spiral was employed (maximum gradient amplitude: 7.6 mT/m, max. slew rate: 174.0 mT/m/ms), requiring 30 interleaves for Nyquist sampling. The FOV increased linearly from 412 mm to 548 mm, with a spatial resolution of 2.5 mm^13^. Fat saturation was applied every 3rd TR to mitigate off-resonance artifacts caused by fatty tissue^23^.

To improve robustness against breathing motion, both Stars and Spirals employed slab-selective excitation and a variable-density pseudo-Cartesian k-space sampling strategy^24^. This approach sampled five central kz-partitions per block while undersampling the outer partitions, achieving a block size of 60 mm with a spatial spacing of 2.5 mm. Acquisition time and signal-to-noise ratio (SNR) were balanced by using undersampling factors of 3 for Stars and 2 for Spirals, tailored to each sequence’s specific requirements.

### Phantom experiment setup

**Fig. 6:**
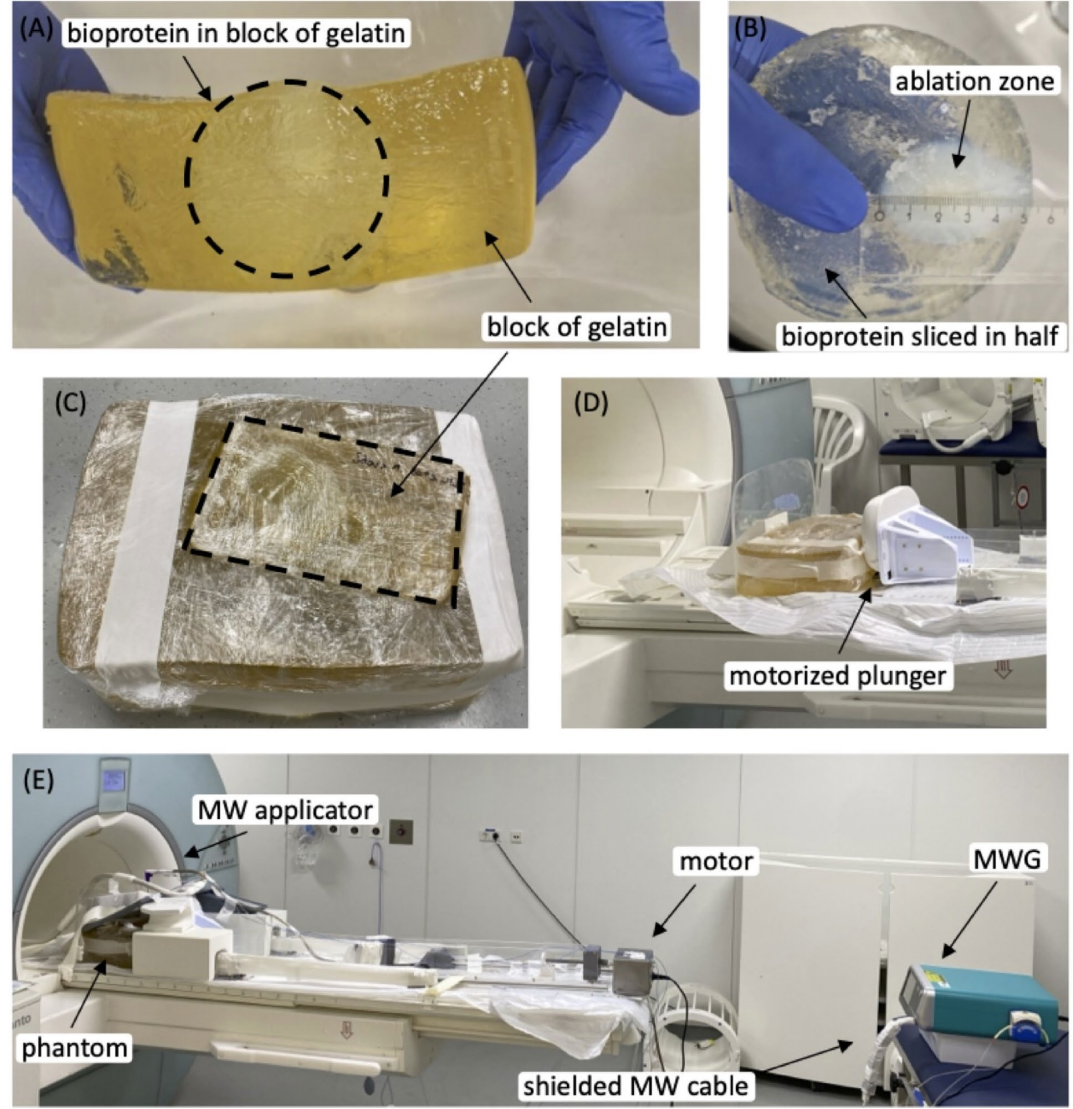
Experimental setup for phantom studies. **(A)** Gelatin phantom with embedded bioprotein phantom. **(B)** Bioprotein phantom sliced in half. **(C)** Complete phantom consisting of a smaller bioprotein-containing gelatin block inserted into a larger gelatin block. **(D)** Gelatin phantom placed in the motorized plunger setup. **(E)** Entire experimental setup including the motorized plunger, gelatin phantom with attached coil, shielded microwave cable, and microwave generator (MWG).

Nine cylindrical bioprotein phantoms (12 cm diameter, 6 cm height) encased in a gelatin block (25.5 × 17.5 × 10 cm³) were used to mimic abdominal dimensions and evaluate both Stars and Spirals sequences^25^. Ablation was performed using a clinically approved microwave generator (MWG, ECO-100E2, ECO Medical Technologies). The experimental setup is depicted in Fig. 6. Although the MWG employs MR-compatible needles and cables, initial scans were disrupted by electromagnetic interference. RF shielding measures, including chokes, copper tape, and copper mesh on the 4 m cable, were implemented, enabling MR thermometry during MWG operation^26^. All safety measures adhered to clinical standards, maintaining medical approval for the device.

The microwave needle was centrally inserted into the bioprotein cylinder. Two fiber optic temperature sensors (FOTEMPTrafo, Weidmann Technologies Deutschland GmbH) were positioned 1.5 to 2.5 cm from the needle tip to provide reference temperatures for MR thermometry evaluation. Breathing motion was simulated using a motorized plunger that compressed the phantom at 11.28 cycles per minute during MR thermometry scans^27^. Each scan consisted of a 3-minute baseline (MWG on standby), a 10-minute ablation at 80 W, and a 3-minute cooling period, resulting in a total scan time of 16 min. After ablation, the plunger was stopped in the decompressed state (exhalation). The ablation zone was assessed with a post-ablation T2-weighted Turbo-Spin-Echo (TSE) sequence (Turbo Factor = 7, FOV = 448 × 210 × 60 mm³, resolution = 1 × 1 × 1 mm³, TE = 156 ms, TR = 10,960 ms). A radiologist segmented these images manually to establish the ground truth for the ablation zone. Segmentations were initially performed in the native T2wTSE space and subsequently interpolated and co-registered using Advanced Normalization Tools to match the geometry of the thermometry data^28^.

### Volunteer study design

Ten healthy volunteers (4 females, 6 males; aged > 18 years) provided informed consent to participate. Volunteer scans followed the same protocol as the phantom experiments, employing a 16-minute free-breathing protocol with identical sequence parameters. The 3D imaging volume was positioned within a central hepatic transverse plane for each subject to ensure consistent and optimal liver coverage.

### Data processing pipeline

Retrospective processing was performed on all acquired data. Gradient delays were calibrated individually for the Stars sequence before each scan, while spiral trajectory calibration was conducted once for all scans^29,30^. The kx–ky plane was aligned with the transverse imaging plane, and the kz direction was oriented head–foot, parallel to B₀ and to the main axis of respiratory motion. Motion correction relied on projection profiles derived from kz-direction surrogate signals of respiratory motion. These profiles were calculated by applying a 1D Fourier transform to central kz-sampling points for each radial projection and spiral interleave^31^. Data from approximately one-third of the respiratory cycle near end-expiration were used for reconstruction. Temporal resolution was evaluated by reconstructing each volume with data combined over 1 and 2 breathing cycles (nBC). For nBC1, the acquisition window matches the typical duration of a single respiratory cycle (~ 5 s), based on individual respiratory timing. Reconstructions utilized compressed sensing and parallel imaging (PICS) with the BART toolbox. Sensitivity maps were generated from low-resolution baseline images^32^. Regularization was applied to improve image quality, including total variation (TV) regularization over 3D space (Spirals: 5 × 10⁻⁴; Stars: 1 × 10⁻⁵), TV over time (Spirals: 5 × 10⁻²; Stars: 5 × 10⁻³), and L2 regularization (Spirals: 5 × 10⁻³; Stars: 1 × 10⁻⁶).

Temporal regularization was applied by incorporating data from the preceding four reconstructed images. Baseline images were reconstructed using PICS without regularization, integrating all data collected during exhalation within the initial 3 min before ablation. Phase drift was estimated through linear regression of phase images in an unheated region of interest (ROI) and corrected by subtracting the global phase drift. Proton resonance frequency shift (PRFS) thermometry was performed by computing phase differences relative to baseline images, with all echoes combined using a weighted sum^33^.

### Ablation zone Estimation

In the phantom experiments, ablation zones were derived from temperature maps using the cumulative equivalent minutes at 43 °C (CEM43) model, applying a 240-minute threshold^34^.

### Data analysis and statistical tests

A range of statistical tests was conducted using Scipy 1.14.1 to validate the results, with statistical significance set at a type I error rate (α) of 0.05 unless otherwise specified.

### Temperature precision in Non-Heated regions

Temperature stability in non-heated regions was evaluated by calculating the standard deviation of temperature values over time within a non-heated 3D region of interest (ROI). The Shapiro-Wilk test was used to assess normality, while Levene’s test determined the homogeneity of variances. Based on normality results, Welch’s t-test or the Mann-Whitney U test was applied to compare temperature accuracy between the Spirals and Stars sequences at nBC1 and nBC2. Additionally, within each sequence, pairwise comparisons between both nBCs were performed using either a paired t-test or a Wilcoxon test, depending on normality.

### Temperature precision in heated regions

Temperature accuracy in heated regions was evaluated by calculating the root mean squared error (RMSE) between MR thermometry measurements and temperature readings from two fiber optic sensors. Sensor positions were visually verified in MR images, and the voxel with the lowest RMSE within a distance of $\:\sqrt{2}$ voxels of the sensor location was selected for analysis^16^. An Analysis of Covariance (ANCOVA) was performed to compare RMSE values between the Stars and Spirals sequences, adjusting for the distance from the ablation center. All ANCOVA assumptions were satisfied. The dependent variable was RMSE (thermometry vs. sensor), the independent variable was the sequence type (Stars vs. Spirals), and the covariate was the sensor’s distance from the ablation center. RMSE differences across nBCs were analyzed using ANOVA, followed by Tukey’s HSD test if significant.

### Accuracy of the calculated ablation zones

Ablation zones from MR thermometry were compared to ground truth zones segmented from T2-weighted TSE images to assess accuracy. Three metrics were used:

- **Dice Score**: Measures the overlap between calculated and ground truth ablation zones. Higher values indicate better overlap: $\:\text{D}\text{S}\text{C}=\frac{2Tp}{2Tp+Fp+Fn}$ where Tp=true positive, Fp=false positive, Fn=false negative.
- **Mean Surface Distance (MSD)**: Reflects the average distance between the surfaces of the calculated and ground truth zones. Lower values indicate better precision: $\:MSD\:=\:\frac{1}{\parallel G \parallel\:+\:\:\parallel S \parallel}\:\left(\sum\:_{s\:\in\:S}\underset{{g\:}\in\:G}{\text{min}}\parallel s\:-g \parallel\:+\sum\:_{g\:\in\:G}\underset{{s\:}\in\:S}{\text{min}}\parallel g-s \parallel\:\right)$, where S=surface points of the predicted zone and G=surface points of the ground truth.
- **Sensitivity**: Represents the proportion of correctly identified ablation, with higher values reflecting improved detection: $\:\text{S}\text{e}\text{n}\text{s}\text{i}\text{t}\text{i}\text{v}\text{i}\text{t}\text{y}=\frac{Tp}{Tp+Fn}$.

Metrics across nBCs were analyzed to evaluate temporal resolution effects. Normality was tested with Shapiro-Wilk, followed by a t-test or Mann-Whitney U based on data distribution. Effect sizes quantified differences between methods. All metrics across nBCs were analyzed with ANOVA, followed by Tukey’s HSD if significant.

### Temperature stability in volunteers scans

In volunteer scans, temperature stability and accuracy were assessed by evaluating the standard deviation of temperature in unheated regions during natural breathing. Three circular regions of interest (ROIs), each 12 voxels in diameter, were manually placed in the liver of each participant, ensuring avoidance of susceptibility artifacts or non-liver structures. ROIs were positioned in caudal, cranial (near the lung diaphragm), and middle slices. Temperature standard deviations for Stars and Spirals sequences across all ROIs were compared using a paired t-test, following normality verification with the Shapiro-Wilk test. If normality was not met, the Wilcoxon signed-rank test was applied. Effect sizes were calculated using Cohen’s d.

### Hardware and reconstruction performance

Reconstruction time was recorded for each nBC. All reconstructions were executed on a high-performance computing server equipped with dual Intel^®^ Xeon^®^ Gold 6342 CPUs (2.80 GHz, 48 cores per processor, 96 threads total), 503 GiB of RAM, and 4 NVIDIA RTX A6000 GPUs (each with 48 GiB VRAM). Despite the availability of multiple GPUs, the BART reconstruction framework utilizes only a single GPU. The system operated on CUDA 12.2 with NVIDIA driver version 535.183.01. The CPU architecture was configured with Non-Uniform Memory Access (NUMA) across two nodes to optimize parallel memory access.

## Supplementary Information

Below is the link to the electronic supplementary material.

Supplementary Material 1

Supplementary Material 2

Supplementary Material 3

Supplementary Material 4

## Data Availability

The datasets generated and/or analyzed during the current study are not publicly available but can be obtained from the corresponding author upon reasonable request. Requests will be considered on a case-by-case basis.
