# Supplementary figures and images for: Evaluation study of radial and spiral based volumetric thermometry for monitoring of hepatic microwave ablation
Source: Sci Rep. 2025 Sep 24;15:32700. doi: 10.1038/s41598-025-20588-4 (PMC12460675; doi:10.1038/s41598-025-20588-4)

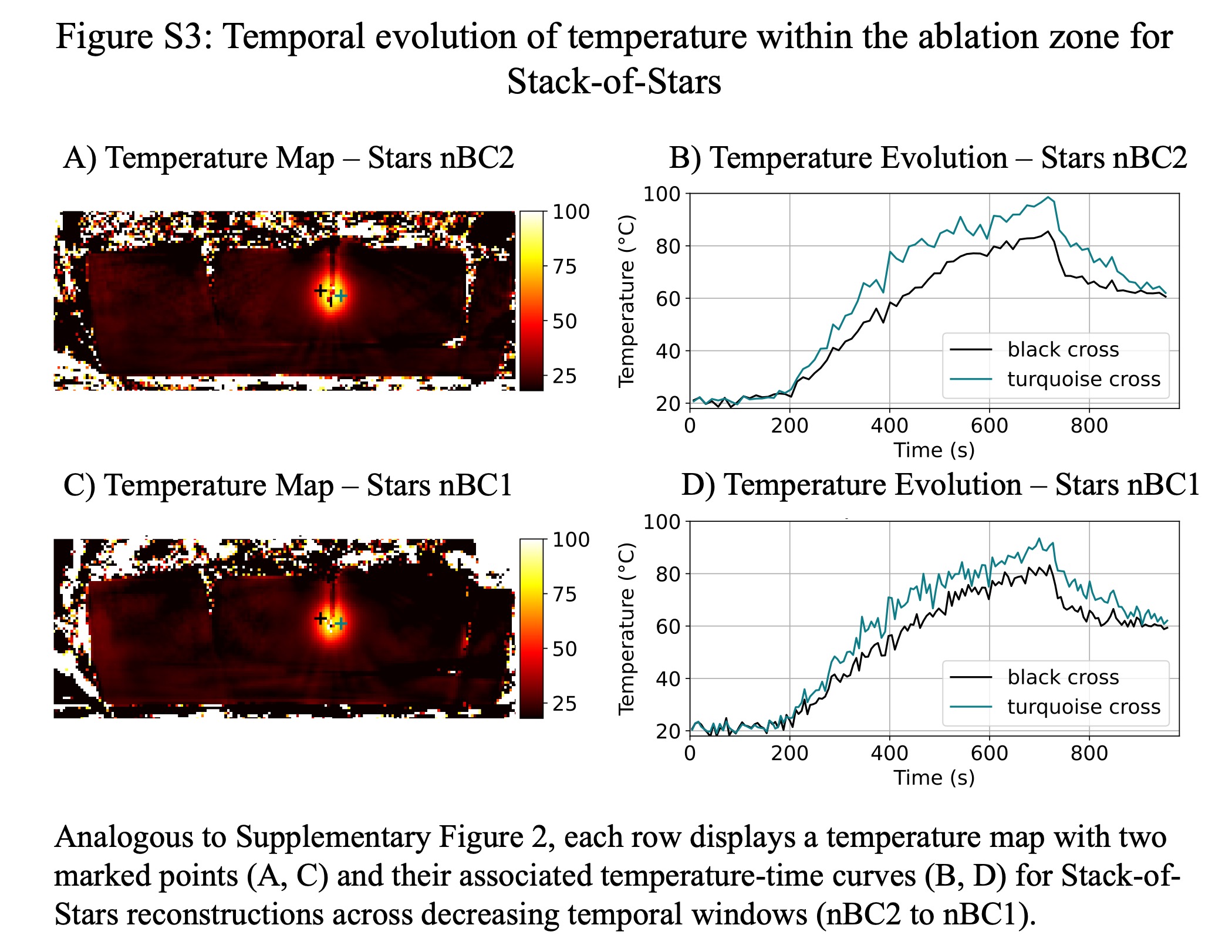

Supplement: Supplementary file 1 — Supplementary Material 1 [file 41598_2025_20588_MOESM1_ESM.jpg]

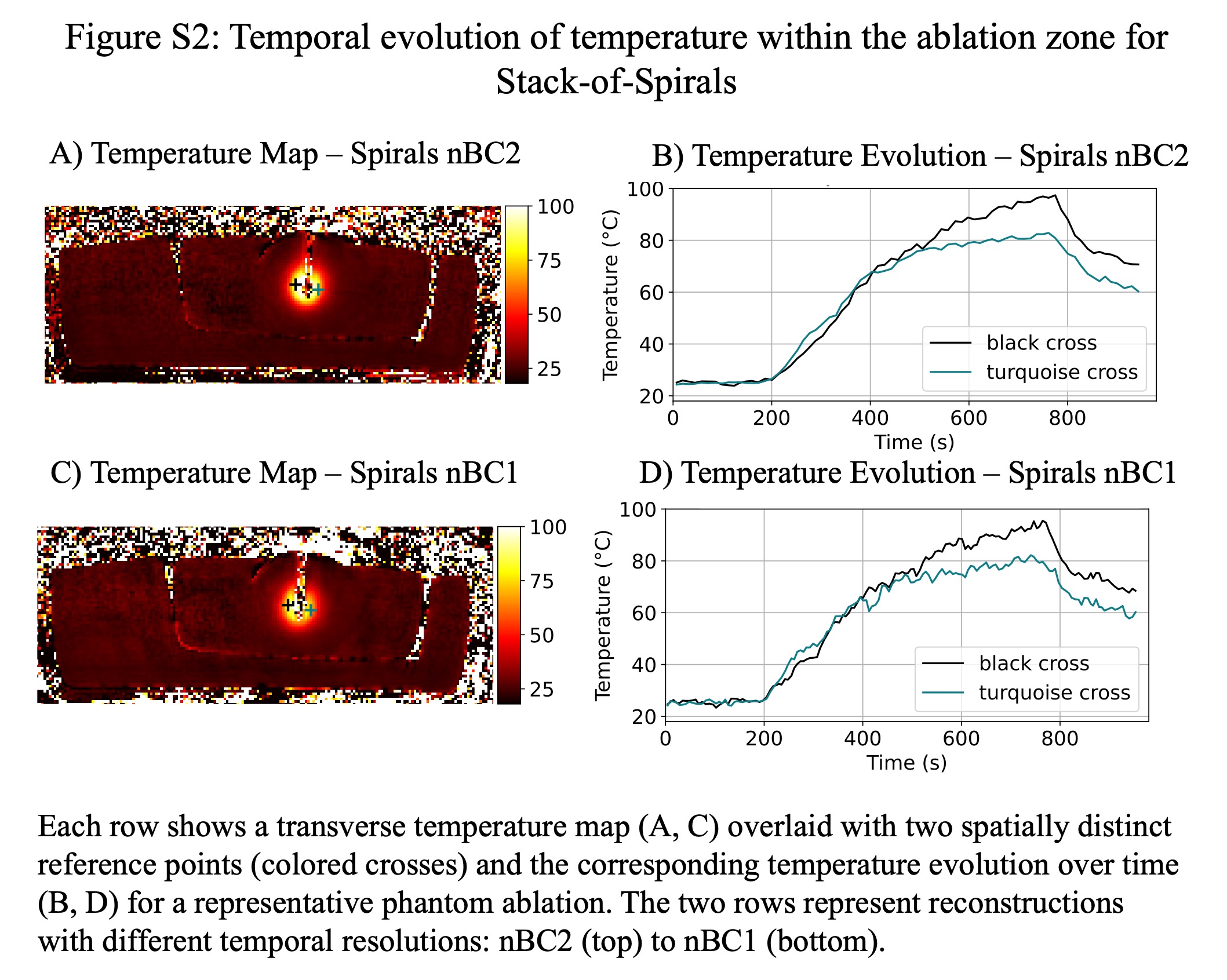

Supplement: Supplementary file 2 — Supplementary Material 2 [file 41598_2025_20588_MOESM2_ESM.jpg]

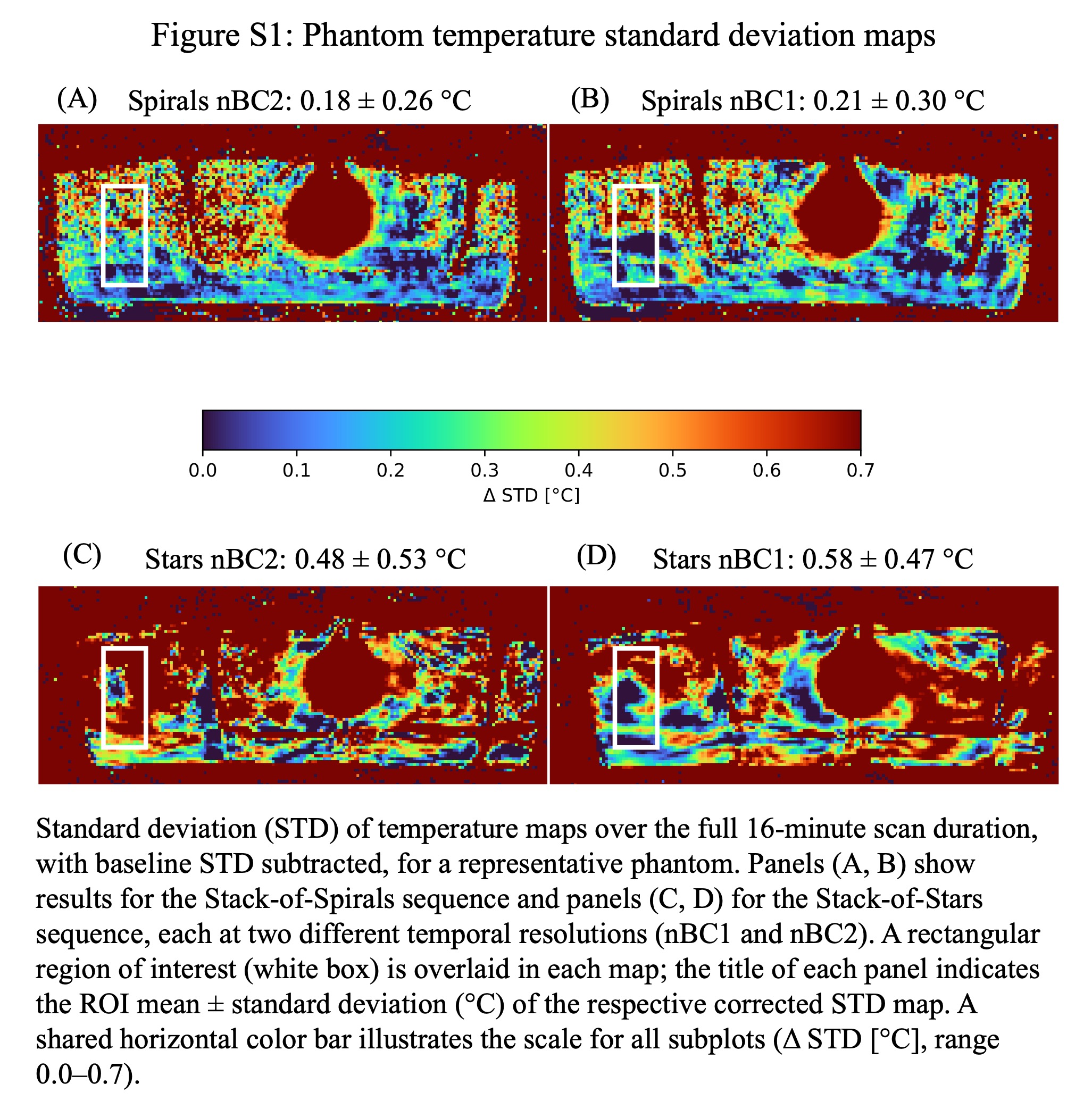

Supplement: Supplementary file 3 — Supplementary Material 3 [file 41598_2025_20588_MOESM3_ESM.jpg]

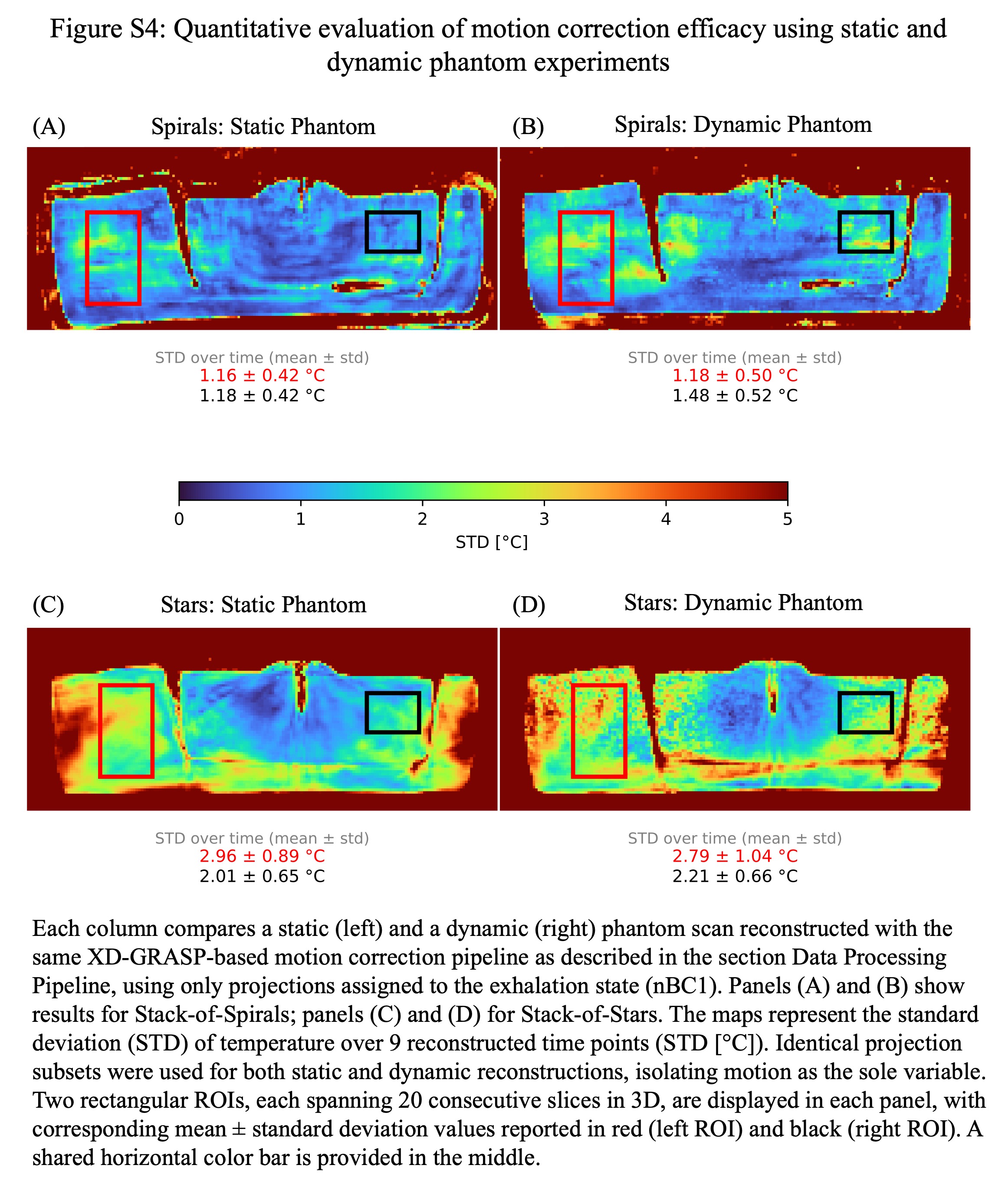

Supplement: Supplementary file 4 — Supplementary Material 4 [file 41598_2025_20588_MOESM4_ESM.jpg]
